# Plant-Parasitic Nematodes Associated with *Cannabis sativa* in Florida

**DOI:** 10.2478/jofnem-2023-0018

**Published:** 2023-07-06

**Authors:** J. Desaeger, J. Coburn, J. Freeman, Z. Brym

**Affiliations:** University of Florida, Department of Entomology and Nematology, Gulf Coast Research and Education Center, Wimauma, FL 33598; University of Florida, Horticultural Sciences Department, North Florida Research and Education Center, Quincy, FL 32351; University of Florida, Department of Agronomy, Tropical Research and Education Center, Homestead, FL 33031.

**Keywords:** *Cannabis sativa*, root-knot nematodes, reniform nematodes, soil type

## Abstract

The subtropical climate of Florida allows for a wide range of crops to be grown. With the classification of hemp (*Cannabis sativa* L., <0.3% delta-9-tetrahydrocannabinol) as an agricultural commodity, hemp has become a potential alternative crop in Florida. Hemp cultivars of different geographies (Europe, China, and North America), and uses (fiber, oil and CBD), were evaluated in three field experiments. The field experiments evaluated a total of 26 cultivars and were conducted for two consecutive seasons at three different locations (soil types) in North (sandy loam), Central (fine sand), and South Florida (gravelly loam). Nematode soil populations were measured at the end of each season. A diverse population of plant-parasitic nematodes was found, with reniform nematodes (RN, *Rotylenchulus reniformis*) the dominant species in North and South Florida (up to 27.5 nematodes/cc soil), and RKN (*Meloidogne javanica*) the main species in central Florida (up to 4.7 nematodes/cc soil). Other nematodes that were commonly found in south Florida (and to a lesser extent north Florida) were spiral (*Helicotylenchus* spp.), stunt (*Tylenchorhynchus* spp.) and ring nematodes (Criconemoids), while in central Florida, stubby root (*Nanidorus minor*) and sting nematodes (*Belonolaimus longicaduatus*) were found. No significant difference among hemp cultivars was noted at any of the locations. RKN were found in all three regions and soils, while RN were only found in North and South Florida. This is the first report on plant-parasitic nematodes associated with hemp in Florida fields. Natural nematode populations varied greatly, depending on where in Florida hemp was grown. Growers who wish to include hemp in their crop rotation need to be aware of potential pest pressure from nematodes. More research is needed to determine to what extent nematodes, especially RKN and RN, can reduce hemp growth and yield.

The subtropical climate of Florida allows for a wide range of crops to be grown, often as an expanded or extreme geographical range. With the removal of hemp (*Cannabis sativa* L., <0.3% delta-9-tetrahydrocannabinol) from the controlled substances list (2018 Farm Bill and 2019 Florida Statute, SB1020 Fla. Stat. § 581.217), hemp now has the potential to be an alternative crop in Florida. There are multiple types of hemp crops harvested for fiber, grain, and cannabidiol, or CBD (Small and Marcus, 2002; Williams, 2019). Different parts of the hemp plant are harvested for each of these specific production targets. Fiber hemp varieties are known to have tall, preferably slender stalks and can grow anywhere from 1 to 5 m in height. Clothing, rope, housing materials, compost, and paper are a few items that can be made from just the stalk of the fiber hemp plant (Cherney and Small, 2016). Fiber hemp products can be used as potentially eco-friendly alternatives or replacements for construction materials and plastics.

Grain hemp cultivars are plants that produce seed that can be used for food items, animal feed, and cosmetics (Cherney and Small, 2016). These plants do not grow as tall as fiber plants, usually averaging 1 to 2 m in height. Dual-use cultivars tend to produce many seeds like grain hemp cultivars, but they also have a stalk that is suitable for use in fiber hemp production. Producers harvesting hemp grain would also harvest the remaining stems for fiber, and this is the most common example of a dual-use hemp crop.

Hemp that is cultivated and bred in order to process flower parts for cannabinoid extracts are usually shorter and have a more brachiate or bushy appearance as compared to fiber and grain. CBD, or cannabidiol, is the non-psychoactive compound commonly extracted from hemp flower and closely resembles THC. CBD can be obtained from the plant by extracting the oil from the flower or by burning or vaporizing the dried flower material. CBD products include topicals, edibles, and smokable products, and is used medicinally to curb pain and muscle spasms (National Academies of Sciences, Engineering, and Medicine, 2017). Cannabinoids are highly concentrated in the trichomes of the bracts and nearby leaves of unfertilized female flowers, much lower in the root and plant tissue, and are at even lower concentrations in hemp pollen and seeds. CBG, or cannabigerol, is another non-psychoactive cannabinoid found in cannabis. Just like CBD hemp, CBG hemp is bred and cultivated for production of the unpollinated female flower.

To support the future viability and sustainability of hemp and considering the importance and prevalence of plant-parasitic nematodes in Florida, it is critical to collect information on the interaction of hemp and nematodes. Plant-parasitic nematodes (PPN) are extremely prevalent in Florida, and many different species are found that can potentially cause damage to many of the crops grown. The most important PPN in Florida in terms of damage potential are root-knot nematodes (*Meloidogyne* spp.), sting nematodes (*Belonolaimus longicaudatus*) and reniform nematodes (*Rotylenchulus reniformis*). All these nematodes have very wide geographic and crop host ranges. Root-knot nematodes are one of the main overall limiting factors to crop production in the world (Sasser, 1980). They are extremely prevalent in Florida, because of the subtropical climate and often sandy soils, with multiple species and races, and a wide host range that includes grasses, vegetables, and fruits. They are the primary nematode problem in Florida vegetables, causing significant losses in tomato, cucurbit, and other vegetables. In Florida strawberries, *M. hapla*, the northern root-knot nematode, has started to become more important as well (Desaeger, 2018). Sting nematodes are also an important soil pest in strawberry, and can cause considerable damage in cantaloupe, potato, sugarcane, forage and turf grasses, citrus and corn, especially in the sandy soils (Smart and Nguyen, 1991; Crow and Brammer, 2001; Noling, 2016). Reniform nematodes also have a very wide host range, including many tropical fruits, ornamentals as well as cotton and soybean (McSorley et al., 1983; Kinlock and Sprenkel, 1994). They are especially common in the Rockdale soils of the southernmost counties in Florida, as well as in the slightly heavier soils of the northwestern Panhandle region (Wang, 2004).

The trials were done during 2019 and 2020 at three different field locations in Florida, (1) the North Florida Research and Education Center (NFREC) in Quincy (North Florida); (2) the Gulf Coast Research and Education Center (GCREC) in Wimauma (West-Central Florida); and (3) the Tropical Research and Education Center (TREC) in Homestead (South Florida) (Table 1). To support the future viability of hemp and considering the importance of PPN in Florida, it is critical to collect information on PPN associated with hemp in Florida.

**Table 1.** Experimental site description.

***Field site***	***County***	***Elevation***	***Soil type***	***Coordinates***
*GCREC^a^ – Central FL*	Hillsborough	31 m	Myakka fine sand	27.76° N, 82.23° W
*TREC^b^ – South FL*	Miami—Dade	1 m	Shallow Krome gravelly loam	25.47° N, 80.50° W
*NFREC^c^ – North FL*	Gadsden	63 m	Orangeburg loamy fine sand	30.58° N, 84.59° W

a GCREC, Gulf Coast Research and Education Center, Wimauma, FL.

b TREC, Tropical Research and Education Center, Homestead, FL.

c NFREC = North Florida Research and Education Center, Quincy, FL.

## Materials and Methods

### Nematode sampling and extraction methodology

Nematode soil samples were taken at the end of each crop/experiment, and roots were visually examined for nematode damage (no preplant samples were collected). Nematode soil samples were taken as four cores per cultivar plot with a locally made cone sampler (3.0 cm-diam. × 25 cm-deep). The four cores were composited for each plot. Soil samples were sealed in plastic bags and were stored at 4°C and nematodes extracted within two weeks. Nematodes were extracted at the GCREC nematology lab by taking a 200 cm^3^ soil sample from each composite sample. A modified Baermann method using a salad spinner (Henrik Preutz, IKEA® USA) was used (Viglierchio and Schmitt, 1983).

Plant-parasitic nematodes (PPN) were identified to genus or species level and by feeding group. Morphological identification was performed using a compound Zeiss AXIO Scope A1 microscope (Carl Zeiss, Göttingen, Germany). Plant-parasitic nematodes (PPN) were identified to genus, and depending on the morphological complexity of the species, the nematode was also identified into species level by using the pictorial key proposed by Mai and Mullin (1996). When root galls were observed on roots, *Meloidogyne* species identification was done by extracting DNA from female nematodes found inside root galls, and mitochondrial haplotype-based identification was done using primers, PCR and RFLP conditions as described by Pagan et al. (2014) and Baidoo et al. (2016). Non-plant-parasitic nematodes (NPPN) nematodes were also morphologically identified into trophic group (bacterivores, fungivores, omnivores).

**Location 1:** Central Florida (GCREC = Gulf Coast Research and Education Center)

Hemp cultivar trials at the Gulf Coast Research and Education Center farm in Wimauma, FL were conducted in October 2019 (season 1, fall) and February 2020 (season 2, spring) in the same location. The soil type in this area is classified as Myakka fine sand (95% sand, <1% organic matter). The experimental area was adjacent to an experimental hops (*Humulus lupulus*) field that had artificial lighting installed by poles with LED lights (GreenPower LED flowering DR/W, Philips) suspended 5.5 m above the ground, staggered 6 m apart (Agehara et al., 2020). Lights provided supplemental light (6 hrs/day) during the first two months of growth which is necessary to extend Florida’s natural day length (max. 14 hrs at GCREC) and create long day conditions required to produce meaningful vegetative growth of hops. Both hemp and hops belong to the Cannabaceae plant family, are dioecious and day-length-sensitive (DLS), with flowering being triggered by short days. The same supplemental lighting regime for hops was applied to hemp, meaning the lights started automatically illuminating an hour before sunset and then turned off at midnight for the first two months after planting. Also, the same fertigation and irrigation schedule was followed in the hemp trials as was done for hops (Desaeger et al., 2022). The experimental area measured 78 m long and 18 m wide and consisted of three rows 78 m long x 1 m wide. Rows were 6 m apart, with bahia grass in between, and were covered with landscape fabric with two lines of drip tape underneath. The drip tape used was Toro BlueLine® with 12″ emitter spacing (0.26 gph, 0.62″ internal diameter, 0.045″ wall thickness; Agehara et al., 2020).

Eight selected hemp cultivars (Table 2) were planted in a randomized block design consisting of four plants of the same cultivar per plot, and each plot was replicated six times (two replicates in each row), for a total of 192 hemp plants. Hemp seedlings were grown in a growth room for two months prior to transplanting in the field. Seeds were sown into 128-cell seedling trays filled with PRO-MIX HP growth medium (Premier Horticulture Inc., Quakertown, PA) on August 16, 2019, and December 29, 2019. Seedlings were grown under supplemental lighting (16-h light and 8-h dark) to maintain vegetative growth. Irrigation was supplied as needed using overhead irrigation. Uniform seedlings of each variety were transplanted to the field on 16 October 2019, and 23 February 2020. Plants measured on average 15–20 cm at the time of transplanting. Watering and fertilizing were administered via in-bed drip tape in both areas. Watering consisted of four one-hour cycles every five hours each day and a soluble fertilizer (N-P_2_O_5_-K_2_O: 5-2-8) was applied with irrigation twice a week throughout the season, based on an accumulated rate of 103 kg N ha^−1^.

**Table 2.** Hemp cultivars with their specific use, country of origin and year(s) planted at each location.

***Cultivars***	***Type/Use***	***Origin***	***Year Planted***
*GCREC^a,b^ and TREC^c^*			
*Yuma-2*	Fiber/Dual	China	2019
*Bama*	Fiber/Dual	China	2019
*Puma-3*	Fiber/Dual	China	2019, 2020
*Tygra*	Fiber/Dual	Poland	2019
*Carmagnola Selezionata*	Fiber/Dual	Italy	2019
*Eletta Campana*	Fiber/Dual	Italy	2019
*Cherry Blossom x T1*	CBD	US	2019
*Cherry Blossom*	CBD	US	2019
***TREC***			
*Berry Blossom*	CBD	US	2019
*Canda*	Fiber/Dual	Canada	2019
*Si-1*	Fiber/Dual	China	2019
*Fibranova*	Fiber/Dual	Italy	2019
*Han-NE*	Fiber/Dual	China	2019, 2020
*Carmagnola*	Fiber/Dual	Italy	2020
*NBS*	CBD	US	2020
*Wife*	CBD	US	2020
*Maverick*	CBD	US	2020
***NFREC^d^***			
*KG 9201*	CBD	US	2019
*KG 9202*	CBD	US	2019
*Cherry Wine*	CBD	US	2019, 2020
*Cherry Blossom x T1*	CBD	US	2019
*Cherry Blossom*	CBD	US	2019, 2020
*Berry Blossom*	CBD	US	2020
*Queens Dream*	CBD	US	2020
*Cinderella Story*	CBD	US	2020
*Cloud Berry*	CBD	US	2020
*Cherry Blonde*	CBD	US	2020
*Hot Blonde*	CBD	US	2020

a GCREC, Gulf Coast Research and Education Center.

b Planting at GCREC was done in fall 2019 and spring 2020, and at TREC and NREC in summer 2019 and summer 2020.

c TREC, Tropical Research and Education Center.

d NFREC, North Florida Research and Education Center.

Both trials were terminated after three to four months, depending on the cultivar.

**Location 2:** South Florida (TREC = Tropical Research and Education Center)

Hemp cultivar trials at TREC in Homestead, FL (25.4687° N, 80.5007° W) were conducted in summer 2019 and summer 2020 on two different fields that were adjacent to each other. The soil type is a shallow Krome, a gravelly loam soil series (loamy-skeletal, carbonatic, hyperthermic, Lithic Rendoll) containing 58% sand, 19% silt, 15% clay, and 8% gravel (Li and Zhang, 2002). Its plowed surface layer, largely crushed bedrock, is 15–20 cm deep with 34% to 76% of limestone fragments (≥ 2 mm in diameter; Bryan and Lance, 1991).

A total of 17 different hemp cultivars (fiber, dual, and CBD) were planted at this location: 13 in 2019 and six in 2020 (Table 2). All cultivars were direct-seeded in the field, and planting densities differed according to their usage. All fiber/dual cultivars were sown by hand in eight rows measuring 1.8 m long and were spaced 30 cm apart within a 5.58 m^2^ (1.83 m × 3.05 m) rectangular plot. For dual cultivars, 900 seeds were planted per plot with an intended planting density of 161 plants/m^2^, while 1500 seeds were planted for fiber cultivars to achieve 269 plants/m^2^. CBD cultivars were planted to establish a density of 11 plants/m^2^ by sowing 60 seeds in the experimental plot and after germination thinning to 11 plants. All plots were fertilized once before planting at 112, 56, and 300 kg/ha of N, P, and K, respectively, using a 6-3-13 slow-release granular fertilizer. Due to early indications of N deficiency, there was an additional N application in mid-July, which was top-dressed using 46-0-0 conventional urea at a rate of 56 kg/ha. Irrigation was provided as needed by overhead sprinklers.

**Location 3:** North Florida (NFREC = North Florida Research and Education Center)

Hemp cultivar trials at NFREC in Quincy, FL were conducted in summer 2019 and summer 2020. Soil type at NFREC is an Orangeburg loamy fine sand. The experimental design was a randomized complete block design with four replications. Three DLS cultivars, including Cherry Blossom (CBL), Cherry × T1 (CT1), and Cherry Wine (CW), and two day-length-neutral (DLN) cultivars, including Kayagene 9201 (KG9201) and Kayagene 9202 (KG9202) were evaluated in 2019. In 2020, cultivars evaluated were CBL, CW, Berry Blossom, Cinderella Story, Cherry Blonde, Cloud Berry, Hot Blonde, and Queens Dream (Table 2). Seeds were sown in the greenhouse using the same methodology as for Location 1, on 14 June 2019 and 2 May 2 2020. Uniform seedlings of each cultivar were transplanted to the field on 3 July 2019 and 12 June 2020.

Agronomic practices were similar between 2019 and 2020. The field setup was similar as for vegetable production in the area, which is plastic-mulch raised beds that were 20-cm high and 30-cm wide with a single drip tape underneath the plastic mulch. The spacing between rows and between plants within a row was 1.8 and 1.5 m, respectively. Therefore, the plant density was ~3600 plants per hectare. Irrigation was supplied daily through the drip tape. Fertilizer (N-P_2_O_5_-K_2_O: 10-10-10) was applied at a rate of 112 kg ha^−1^ immediately prior to transplanting and disked into soils. A soluble fertilizer (N-P_2_O_5_-K_2_O: 4-0-8) was applied with irrigation as needed throughout the season based on an accumulated rate of 56 kg N ha^−1^.

## Results

At the GCREC, by the end of the first season, stubby root nematodes (*Nanidorus minor*) and NPPN (bacterivorous, fungivorous, and omnivorous) were found (Table 3). Nematode populations in the soil were higher in the second season. By the end of season two, stubby root nematodes, root-knot nematodes (*Meloidogyne javanica*), as well as sting nematodes (*Belonolaimus longicaudatus*) were found. No significant difference was noted between cultivars for either root-knot, sting or stubby root nematodes (Table 3). No root galls were noted in the first season, and few small root galls were seen on two cv. Cherry Blossom plants in season 2.

**Table 3.** Nematode soil populations (no./200 cc soil) at harvest of eight different hemp cultivars for two consecutive seasons, GCREC, Fall 2019 (trial 1) and Spring 2020 (trial 2)^a^.

***Sept. – Dec. 2019 (Fall)***	***Bacterivore***	***Fungivore***	***Omnivore***	***Stubby root***	***Root-knot***	***Sting***
*Yuma-2*	49	31	12	31 a	0	0
*Bama*	117	18	7	21 ab	0	0
*Puma-3*	120	32	12	32 a	0	1
*Tygra*	70	30	7	6 b	0	0
*Carmagnola Selezionata*	121	30	9	12 ab	0	1
*Eletta Campana*	89	19	7	11 ab	0	0
*Cherry Blossom x T1*	121	32	5	23 ab	0	1
*Cherry Blossom*	72	25	7	15 ab	0	1
*P* value	*0.24*	*0.75*	*0.31*	*0.02*	-	-

***Feb.-May 2020 (Spring)***	***Bacterivore***	***Fungivore***	***Omnivore***	***Stubby root***	***Root-knot***	***Sting***
*Yuma-2*	-	-	-	18 abc	16	15
*Bama*	-	-	-	62 ab	437	3
*Puma-3*	-	-	-	62 ab	610	13
*Tygra*	-	-	-	11 bc	289	14
*Carmagnola Selezionata*	-	-	-	11 bc	9	16
*Eletta Campana*	-	-	-	8 c	117	9
*Cherry Blossom x T1*	-	-	-	65 a	100	8
*Cherry Blossom*	-	-	-	24 abc	315	32
*P* value	-	-	-	*0.002*	*0.16*	*0.17*

a Numbers listed describe the number of a type of nematode found in 200 cc of soil collected from the root area of the hemp cultivars listed. There are multiple types/uses of hemp here. *P* values were calculated on Log (x+1) transformed data according to Tukey’s HSD where *P* ≤ 0.05; bacterivores, fungivores, omnivores were not counted in the second season.

Nematode soil populations in the hemp trials at TREC consisted of several PPN: reniform nematodes (*Rotylenchulus reniformis*), stunt nematodes (*Tylenchorhynchus* spp.), spiral nematodes (*Helicotylenchus* spp.), ring nematodes (*Criconemella* spp. or *Mesocriconema* spp.), and root-knot nematodes (*Meloidogyne* spp.), as well as NPPN (Table 4). The highest reniform nematode populations were found in the first trial on the fiber cultivars Puma and Carmagnola Selezionata (*P* < 0.004). The second trial was done on an adjacent field with mostly CBD-type hemp cultivars. In this trial, reniform, ring and root-knot nematodes were found (Table 4). No significant differences for any of the nematodes were observed between the tested cultivars in season 2, and no root galls were observed on any of the roots.

**Table 4.** Nematode soil populations (no./200 cc soil) at harvest of different hemp cultivars for two consecutive seasons, summer 2019 and summer 2020; TREC soil survey results^a^.

***Summer 2019***	***Bacterivore***	***Fungivore***	***Omnivore***	***Reniform***	***Stunt***	***Spiral***	***Ring***	***RKN***
*Puma-3*	116	42	0	5,564 a	112	176	12	0
*Bama*	70	74	0	1,987 ab	156	221	26	0
*Berry Blossom*	28	52	0	574 abc	32	92	2	0
*Tygra*	22	26	2	762 abc	8	26	14	0
*Carmagnola Selezionata*	52	100	8	4,872 a	80	84	12	0
*Helena*	68	48	2	648 abc	24	48	0	0
*Canda*	80	116	8	480 abc	40	54	6	0
*Si-1*	54	21	0	319 c	178	286	0	0
*Fibranova*	83	27	0	333 bc	15	373	0	0
*Yuma-2*	39	29	2	636 abc	119	524	1	0
*Han NE*	25	12	1	195 c	11	266	0	0
*Cherry Blossom x T1*	42	25	0	989 abc	38	119	0	0
*Eletta Campana*	36	52	1	844 abc	30	216	14	0
*P* value	*0.06*	*0.04*	*0.05*	*0.004*	*0.20*	*0.19*	*0.19*	*-*

***Summer 2020***	***Bacterivore***	***Fungivore***	***Omnivore***	***Reniform***	***Stunt***	***Spiral***	***Ring***	***RKN***
*Puma-3*	141	31	1	78	79	148	73	41
*Carmagnola*	173	43	1	101	81	109	65	74
*Han NE*	180	42	4	214	216	409	17	62
*NBS*	73	19	2	22	54	256	59	20
*Wife*	215	50	2	69	108	686	447	57
*Maverick*	111	32	1	32	53	289	35	18
*P* value	*0.21*	*0.22*	*0.82*	*0.15*	*0.11*	*0.06*	*0.25*	*0.57*

a Numbers listed describe the number of a type of nematode found in 200 cc of soil collected from the root area of the hemp cultivars listed. There are multiple types/uses of hemp here. *P* values were calculated on Log (x+1) transformed data according to Tukey’s HSD where *P* ≤ 0.05; RKN = root-knot.

At NFREC, nematode soil populations consisted of the same PPN as at TREC, although at lower numbers (Table 5). Reniform nematodes (*Rotylenchulus reniformis*) were the most common, and low populations of stunt nematodes (*Tylenchorhynchus* spp.), spiral nematodes (*Helicotylenchus* spp.), ring nematodes (*Criconemella* spp. or *Mesocriconema* spp.), and root-knot nematodes (*Meloidogyne incognita*) were found (Table 5). Only CBD cultivars were planted at this location, and no significant differences were noted among cultivars. Few root galls were observed on cv. Cherry Blossom in the second season.

**Table 5.** Nematode soil populations (no./200 cc soil) at harvest of different hemp cultivars for two consecutive seasons, summer 2019 and summer 2020; NFREC soil survey results^a^.

***Summer 2019***	***Bacterivore***	***Fungivore***	***Omnivore***	***Reniform***	***Stunt***	***Spiral***	***Ring***	***RKN^b^***
*KG 9201*	139	40	0	41	1	0	0	3
*Cherry Wine*	158	47	0	92	4	2	0	5
*KG 9202*	312	74	1	127	3	0	1	4
*Cherry Blossom x T1*	307	80	0	239	1	6	0	65
*Cherry Blossom*	304	68	1	191	2	3	0	16
*P value*	*0.42*	*0.30*	*0.54*	*0.43*	*0.71*	*0.36*	*0.44*	*0.33*

***Summer 2020***	***Bacterivore***	***Fungivore***	***Omnivore***	***Reniform***	***Stunt***	***Spiral***	***Ring***	***RKN***
*Berry Blossom*	309	52	0	67	0	2	0	9
*Queens Dream*	212	32	1	40	2	0	0	3
*Cinderella Story*	119	61	0	103	1	2	0	38
*Cherry Blossom*	441	86	0	328	2	4	0	4
*Cloud Berry*	320	75	2	101	3	0	2	33
*Cherry Blonde*	167	50	0	53	1	1	0	41
*Hot Blonde*	319	115	0	453	1	9	0	28
*Cherry Wine*	224	64	1	90	2	1	0	83
*P value*	*0.95*	*0.96*	*0.19*	*0.83*	*0.76*	*0.34*	*0.62*	*0.17*

a Numbers listed describe the number of a type of nematode found in 200 cc of soil collected from the root area of the hemp cultivars listed. There are only CBD hemp cultivars here. *P* values were calculated on Log (x+1) transformed data according to Tukey’s HSD where *P* ≤ 0.05.

b RKN, root-knot.

Bacterivorous, fungivorous, and omnivorous nematodes (NPPN) were prevalent at all three locations and showed no difference between cultivars or seasons.

## Discussion

Seven different PPN were found on hemp in Florida. Most common were root-knot nematodes, which were found at all three locations, and reniform nematodes, which were found in North (NFREC) and South Florida (TREC).

Root-knot nematodes were the dominant nematode in Central Florida (GCREC) after the second season, despite the fact that none were found in the same field at the end of the first season. This shows our limited ability to quantify nematode populations and how difficult it can be to detect the presence of root-knot in a newly planted field. Probably, the deep sand soils that are common in Central Florida harbor significant amounts of root-knot inoculum in its deeper layers (Noling, 2019). It also indicates that in the presence of a good host, root-knot nematodes can build up rapidly in the subtropical climate of Florida. Recent reports from the US and China have shown that hemp can be a good host to various root-knot nematodes, including *M. incognita, M. hapla*, and *M. enterolobii* (Song et al., 2017; Kotcon et al., 2018; Ren et al., 2020; Lawaju et al., 2021; Bernard et al., 2022; Coburn et al., 2022). No noticeable root damage symptoms were seen on any of the cultivars, except at GCREC and NFREC on a few plants from cv. Cherry Blossom that showed small but visible root galls.

Stubby root nematodes (*Nanidorus minor*) were the only PPN found in the first season at the GCREC, with slightly higher numbers in the second season. Sting nematodes (*Belonolaimus longicaudatus*), like root-knot, only showed up in the second season. Both stubby root and sting are ectoparasitic nematodes and very common in the sandy soils of Florida and were only found at the GCREC. They can cause considerable damage to a wide range of crops, such as potato, strawberry, vegetables, corn, citrus, and others (Crow and Brammer, 2001; Noling, 2016). Reniform nematodes were only found at TREC and NFREC and were most common on the fiber cultivars Puma and Carmagnola at TREC in 2019. Reniform nematodes are semi-endoparasitic nematodes that live and feed somewhat immersed within plant roots, with the tail end of the female body protruding into the soil (Wang, 2004). There are around 300 plant species that are known hosts to reniform nematodes. In the southern part of Florida where TREC is located, reniform nematode is a pest to many tropical fruits (McSorley et al., 1983). Additionally, in the northern part of Florida where NFREC is located, reniform nematode is known to be a major nematode pest in cotton (Wang, 2004). Finding this nematode in both locations can be cause for concern due to the types of crops grown in those areas of Florida.

Cultivar differences were limited, and none of the 26 cultivars that were planted across the different locations showed a consistent response. More controlled experiments are needed to verify potential cultivar and genetic differences with regard to their host potential to root-knot and other PPN. This will allow us to tell which cultivars are best suited for which location. At TREC, hemp cv. Wife, which was previously reported to be resistant to the RKN species *M. incognita* (Bernard et al., 2022), had root-knot nematode counts similar to other hemp cultivars. Possibly, this was due to root-knot nematodes in the field trial reproducing on weeds in these plots (which were abundant but not identified), or the root-knot nematode in the field may have been a different species, race, or population. We did not identify the root-knot nematode species at TREC, but previous studies at this location identified the RKN species at this field as *M. incognita* (Zhang et al., 2022).

The above results show that many different PPN can be associated with hemp in Florida, and that populations will vary by region. In North Florida, root-knot and reniform nematodes were the most common. In South Florida, in addition to reniform and root-knot, also spiral, stunt, and ring nematodes were abundant. In Central Florida, root-knot, sting, and stubby root nematodes were found. These regional differences in nematode populations are likely correlated with the different soil types. Sting and stubby root nematodes seem to prefer the almost pure sandy soils in Central Florida, while reniform and spiral nematodes are more common in North and South Florida, where soils are less sandy, having a higher loam and silt content.

Root-knot nematodes are of most concern due to their statewide distribution and high damage potential to many of the crops grown in Florida. A literature review by Bernard et al. (2022) on nematode interactions with hemp also reported that *Meloidogyne* was the most reported genus. No differences were noted in nematode populations for the different cultivars, but more research is needed to study this in more detail and under more controlled conditions. In addition, there is also a need to study to what extent especially root-knot and reniform nematodes can impact hemp production in the state.

Hemp production is still very new in Florida and for it to become profitable much more research will be needed. The lack of an earlier soil sample date that would enable comparisons between transplanting and harvest is a weakness in this study and cannot be corrected. Nevertheless, this study provides a “first look” and gives new information on which PPN are associated with different hemp types and cultivars in three different regions of Florida. It is a first step, and it is hoped that this research can form a basis for more focused nematode research in terms of nematode management. Such information would be very valuable to current and future hemp growers in Florida.
